# Recurrent Pneumococcal Meningitis Without Cerebrospinal Fluid Pleocytosis and With Fulminant Septic Shock: A Case Report

**DOI:** 10.7759/cureus.116945

**Published:** 2026-09-25

**Authors:** Daisuke Shiina, Koichi Zokumasu

**Affiliations:** 1 Emergency Medicine, Kanto Central Hospital, Tokyo, JPN; 2 Gastroenterology, National Center for Global Health and Medicine, Tokyo, JPN

**Keywords:** adult bacterial meningitis, emergency medicine, normal cerebrospinal fluid findings, pneumococcal meningitis, recurrent meningitis

## Abstract

Bacterial meningitis is typically diagnosed via lumbar puncture. It usually occurs with cerebrospinal fluid (CSF) abnormalities. This report describes the case of a 68-year-old man with recurrent pneumococcal meningitis and absence of significant CSF pleocytosis. Despite a thorough search, no infectious focus was identified. The patient lacked typical meningeal signs, such as neck stiffness, and did not initially present with marked alteration of consciousness. Prompt antibiotic therapy was initiated; however, the patient died on the day of admission. On the day after his death, Gram-positive diplococci were detected in blood, urine, and CSF cultures and were subsequently identified as *Streptococcus pneumoniae*. The CSF examination showed no findings strongly suggestive of meningitis.

Atypical presentations of meningitis may lead to delays in diagnosis and treatment. To the best of our knowledge, reports of such cases are rare.

## Introduction

Bacterial meningitis affects approximately 1,700 individuals annually in Japan [1]. It remains a critical medical emergency, with a mortality rate of approximately 20%, while nearly half of survivors experience long-term sequelae, such as hearing loss [2]. Clinically, the disease is characterized by the classic triad of fever, neck stiffness, and loss of consciousness [3]. Lumbar puncture is essential for diagnosis, typically revealing findings such as neutrophil-dominant pleocytosis, elevated protein levels, and a decreased cerebrospinal fluid (CSF)-to-serum glucose ratio [4]. However, it is recognized, albeit rare, that some cases of bacterial meningitis present without CSF pleocytosis. Here, we report a rare and instructive case of recurrent pneumococcal meningitis characterized by the absence of CSF pleocytosis and a fulminant clinical course. Bacterial meningitis without CSF pleocytosis is rare, and recurrent cases are particularly uncommon, with only a limited number of cases reported in the literature. The accumulation and analysis of similar cases are essential. Therefore, we report this case to contribute to the limited literature on this clinical presentation.

## Case presentation

A 68-year-old man with a medical history of ascending thoracic aortic aneurysm replacement and prior pneumococcal meningitis presented to our internal medicine outpatient department with bilateral upper extremity pain. He had been in his usual state of health and was able to play golf until four days before presentation. Two days before admission, he developed generalized pain in both his upper and lower extremities, followed by the onset of dyspnea the night before presentation. On arrival, he appeared cyanotic and was immediately transferred to the emergency department.

The patient had no regular medications or known allergies. His alcohol consumption was approximately 20 g of ethanol three times per week, and he had a smoking history of 30 cigarettes per day until eight years prior. He had previously worked in the construction industry and lived in Southeast Asia. He had received the pneumococcal vaccine at age 65.

On physical examination, prominent livedo reticularis was observed over the patient’s entire body, although no other specific findings were noted. Heart sounds were normal, and there were no signs of meningeal irritation, such as neck stiffness. His level of consciousness was Japan Coma Scale (JCS) 1 [5], body temperature was 38.2°C, blood pressure was 130/105 mmHg, pulse rate was 135 beats/minute, respiratory rate was 30 breaths/minute, and SpO₂ was 92% while receiving oxygen at 10 L/min via a reservoir mask.

Laboratory findings revealed a white blood cell count of 12,400/µL, hemoglobin of 14.6 g/dL, and platelets of 4.3 × 10⁴/µL. Serum biochemistry showed creatinine of 3.32 mg/dL, C-reactive protein of 23.2 mg/dL, and procalcitonin of 81.31 ng/mL. Hepatic enzymes were elevated (aspartate aminotransferase 142 IU/L, alanine aminotransferase 72 IU/L), and the total bilirubin level was 1.6 mg/dL. Coagulation tests revealed a prothrombin time-international normalized ratio of 1.54, an activated partial thromboplastin time of 55.1 seconds, and a D-dimer of 275.59 µg/mL. Electrolytes and urinalysis results were within normal limits (Table 1).

**Table 1:** Laboratory data

Parameter	Patient value	Reference range
White blood cell, /µL	12,400	3,500-9,500
Red blood cell, 10⁴/µL	462	480-600
Hemoglobin, g/dL	14.6	14.0-18.0
Platelet count, 10⁴/µL	4.3	13.0-40.0
Aspartate aminotransferase, U/L	142	8-38
Alanine aminotransferase, U/L	72	4-44
Gamma-glutamyl trans peptidase, U/L	26	13-64
Alkaline phosphatase, U/L	72	30-130
Total bilirubin, mg/dL	1.6	0.2-1.2
Direct bilirubin, mg/dL	0.5	0.1-0.4
Lactate dehydrogenase, U/L	639	125-243
Blood urea nitrogen, mg/dL	35	8-20
Creatinine, mg/dL	3.32	0.60-1.10
Sodium, mEq/L	136	135-147
Potassium, mEq/L	3.8	3.3-4.8
Glucose, mg/dL	67	70-119
C-reactive protein, mg/dL	23.2	0.00-0.30
Procalcitonin, ng/mL	81.31	0.00-0.50
Activated partial thromboplastin time, seconds	55.1	23.7-34.8
Prothrombin time-international normalized ratio	1.54	0.85-1.15
D-dimer, µg/mL	275.59	0.0-1.0

Arterial blood gas analysis revealed a serum lactate level of 8.2 mmol/L, raising concern for severe infection. Contrast-enhanced CT of the trunk and head was performed to identify a potential source of infection; however, no significant abnormalities were detected. Arterial blood gas analysis showed a partial pressure of oxygen (PaO₂) of 371 mmHg and a partial pressure of carbon dioxide (PaCO₂) of 23.9 mmHg. Although the assessment of oxygenation was limited given that the patient was receiving oxygen at 10 L/min via a reservoir mask, there was no evidence of overt hypoxemia. The findings also indicated high-anion-gap metabolic acidosis, predominantly attributable to lactic acidosis, with concomitant respiratory alkalosis in the setting of severe systemic illness (Table 2).

**Table 2:** Arterial blood gas analysis

Parameter	Patient value	Reference range
pH	7.375	7.35-7.45
Partial pressure of oxygen (PaO₂), mmHg (receiving oxygen at 10 L/min)	371	80-100
Partial pressure of carbon dioxide (PaCO₂), mmHg	23.9	35-45
Bicarbonate (HCO₃), mmol/L	14	22-26
Anion gap, mmol/L	24.1	10-14
Lactate, mmol/L	8.2	0.5-1.5

Owing to the patient's altered consciousness, we performed brain MRI, which revealed multiple cerebral infarctions in both cerebellar hemispheres and the right frontal lobe (Figures 1, 2). 

**Figure 1:**
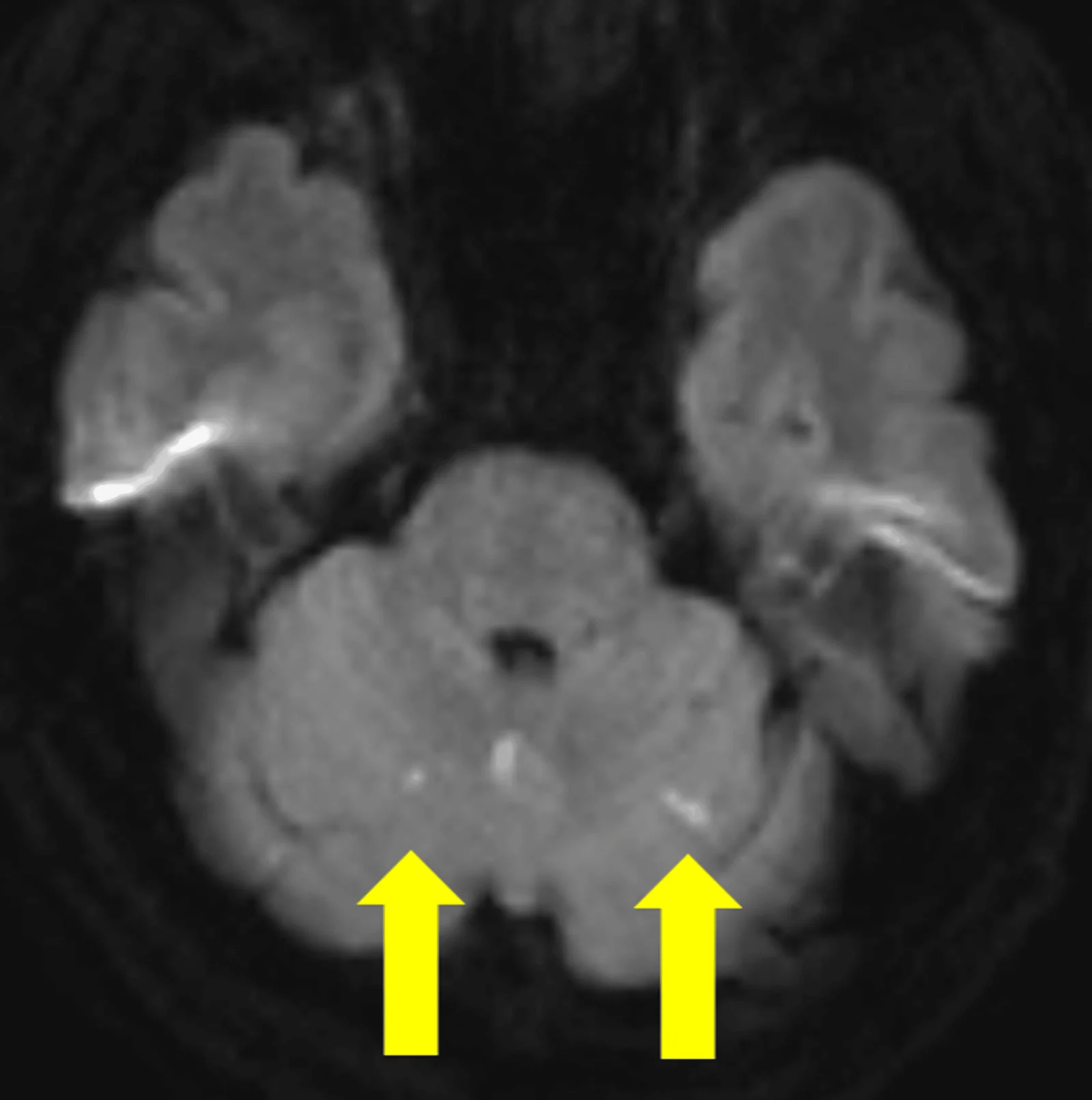
Diffusion-weighted imaging (DWI) showing a hyperintense lesion in the cerebellum After presentation, the patient developed impaired consciousness. Brain MRI was performed, and DWI revealed multiple scattered hyperintense lesions in both cerebellar hemispheres. The arrows indicate the hyperintense lesion.

**Figure 2:**
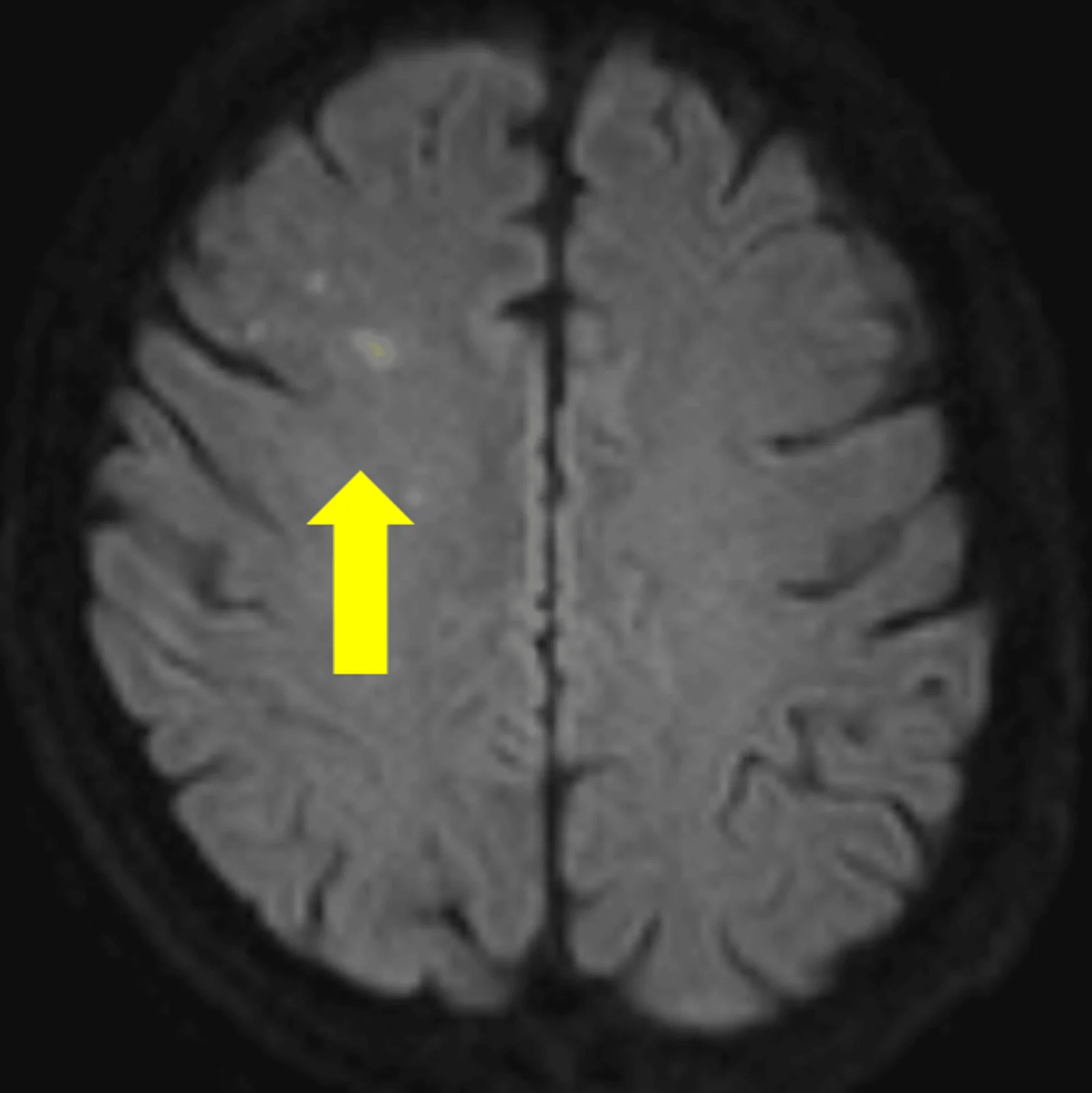
Diffusion-weighted imaging (DWI) showing a hyperintense lesion in the right frontal lobe. After presentation, the patient developed impaired consciousness. Brain MRI was performed, and DWI revealed a hyperintense lesion in the right frontal lobe. The arrow indicates the hyperintense lesion.

These lesions did not conform to specific vascular territories. After consultation with a neurologist and a radiologist, the lesions were considered to represent septic emboli. Although we performed transthoracic echocardiography, no obvious vegetations were identified, and infective endocarditis was not strongly suspected. We performed a lumbar puncture; however, the CSF was colorless and transparent, with lymphocytes 3/µL, neutrophils <1/µL, and glucose 38 mg/dL (serum glucose 67 mg/dL). These findings were initially considered inconsistent with meningitis (Table 3).

**Table 3:** Cerebrospinal fluid (CSF) values

Parameter	Patient value	Reference range
Opening pressure, cmH_2_O	15	5-18
Glucose level, mg/dL	38	50-75
Protein, mg/dL	65	15-45
Leukocyte count, x 10⁶/L	3	<5.0
Neutrophil count, x10⁶/L	<1.0	<5.0
Ratio of CSF glucose level to serum glucose level	0.57	0.60-0.80
Appearance	Crystal clear	
Gram stain (Identified the following day)	Gram-positive Cocci	
Culture (Identified the following day)	Streptococcus pneumoniae	

The patient was admitted to the intensive care unit (ICU) with a diagnosis of sepsis of unknown origin. We administered broad-spectrum antimicrobial therapy with meropenem (1 g/day) and vancomycin (1 g/day) when the patient was admitted to the ICU. The doses were determined based on the patient's renal function. Blood and urine samples for culture were collected at the time of the initial laboratory evaluation.

One hour after ICU admission, the patient developed hypotension and was administered norepinephrine (0.05 µg/kg/min). Two hours after ICU admission, the patient developed apnea and was intubated. Despite the addition of vasopressin (0.05 µg/kg/min) and hydrocortisone (200 mg), his condition rapidly deteriorated, and he died seven hours after ICU admission.

On the following day, Gram-positive diplococci were detected in blood, urine, and CSF cultures, confirming the diagnosis of pneumococcal meningitis. Susceptibility testing was not performed because the patient died. 

An autopsy performed the following day revealed whitish, opaque meninges over the parietal lobes, although there was no evidence of abscess formation.

No evidence of pneumonia, deep vein thrombosis, or pulmonary embolism was found. The middle ear and paranasal sinuses were not examined at autopsy. The adrenal glands showed no petechial hemorrhages; Waterhouse-Friderichsen syndrome was considered unlikely.

## Discussion

This case represents a rare and fatal instance of recurrent pneumococcal meningitis, characterized by the absence of CSF pleocytosis and a fulminant clinical course, resulting in septic shock. CSF analysis obtained by lumbar puncture plays a central role in the diagnosis of bacterial meningitis. Typical findings include neutrophil-dominant pleocytosis, elevated protein levels, and a decreased CSF-to-serum glucose ratio [4,6]. However, cases exist in which bacteria are identified through PCR or culture despite the absence of CSF pleocytosis [6,7].

Although bacterial meningitis without CSF pleocytosis has been reported in 0.5%-12% of pediatric cases, it is considered exceedingly rare in immunocompetent adults [8]. Previous studies have estimated the incidence of bacterial meningitis without CSF pleocytosis at approximately 1.7% [9]. Several factors may contribute to the absence of CSF pleocytosis, including antimicrobial administration prior to lumbar puncture, hematogenous spread from other sites, underlying immunosuppressive states such as diabetes, alcohol dependence, asplenia, use of immunosuppressive agents, or malignancy, as well as hyperacute disease progression [9,10]. In the present case, no prior antibiotics were administered, and there was no evidence of an immunocompromised state. Therefore, the precise reason for the absence of a cellular response in the CSF remains uncertain.

Another salient feature of this case is the recurrence of pneumococcal meningitis, occurring 13 years after the initial episode. Recurrent meningitis occurs in approximately 5% of patients with meningitis [11]. It is defined as two or more episodes caused by different pathogens or reinfection by the same pathogen after appropriate treatment of the initial infection [12]. Recurrence is frequently associated with anatomical defects (either congenital or acquired through trauma or surgery), chronic infections of the middle ear or paranasal sinuses, CSF rhinorrhea/otorrhea, or immunodeficiency. Although our autopsy did not identify a clear skull base deformity, the middle ear and paranasal sinuses were not examined at autopsy. Therefore, an underlying anatomical predisposition could not be definitively excluded, which represents a limitation of this case. In addition, data regarding the pneumococcal serotype during the initial episode and the type of pneumococcal vaccine administered were unavailable. These limitations preclude a definitive assessment of the factors predisposing this patient to recurrent pneumococcal meningitis.

Notably, recurrent meningitis typically has a lower mortality rate than non-recurrent forms, and in 43% of recurrent cases, no definitive predisposing factor is identified [11,12]. While Cergole-Novella reported a fatal case of recurrent pneumococcal meningitis following traumatic brain injury with a CSF leak [13], most recurrent cases have a favorable prognosis because symptoms such as otorrhea or rhinorrhea often lead to earlier clinical presentation and intervention. Thus, the fatal outcome in our patient was atypical for recurrent meningitis, particularly given that his initial episode 13 years earlier was accompanied by CSF pleocytosis typical of bacterial meningitis.

Furthermore, although the classic triad of fever, neck stiffness, and altered mental status is well-known [3], all three signs are present in only 44% of cases [11]. In particular, fulminant meningitis, which can result in death within hours, may lack clear meningeal signs [14]. In this case, the clinical presentation at admission did not strongly suggest meningitis.

## Conclusions

In conclusion, this case demonstrates that pneumococcal meningitis may follow a fulminant and fatal course even in the absence of CSF pleocytosis and classic meningeal signs. The fulminant clinical course raises the possibility of a hyperacute process; however, the reason for the limited CSF cellular response remains uncertain. Bacterial meningitis should remain in the differential diagnosis even in the absence of CSF pleocytosis or classic meningeal signs. Given the limited number of reported cases, further accumulation and analysis of similar cases are warranted.
